# Understanding the formation mechanism of lipid nanoparticles in microfluidic devices with chaotic micromixers

**DOI:** 10.1371/journal.pone.0187962

**Published:** 2017-11-28

**Authors:** Masatoshi Maeki, Yuka Fujishima, Yusuke Sato, Takao Yasui, Noritada Kaji, Akihiko Ishida, Hirofumi Tani, Yoshinobu Baba, Hideyoshi Harashima, Manabu Tokeshi

**Affiliations:** 1 Division of Applied Chemistry, Faculty of Engineering, Hokkaido University, Kita-ku, Sapporo, Japan; 2 Graduate School of Chemical Sciences and Engineering, Hokkaido University, Kita-ku, Sapporo, Japan; 3 Faculty of Pharmaceutical Sciences, Hokkaido University, Kita-ku, Sapporo, Japan; 4 Department of Applied Chemistry, Graduate School of Engineering, Nagoya University, Chikusa-ku, Nagoya, Japan; 5 ImPACT Research Center for Advanced Nanobiodevices, Nagoya University, Chikusa-ku, Nagoya, Japan; 6 Institute of Innovation for Future Society, Nagoya University, Chikusa-ku, Nagoya, Japan; 7 Innovative Research Center for Preventive Medical Engineering, Nagoya University, Chikusa-ku, Nagoya, Japan; Chung-Ang University College of Engineering, REPUBLIC OF KOREA

## Abstract

Lipid nanoparticles (LNPs) or liposomes are the most widely used drug carriers for nanomedicines. The size of LNPs is one of the essential factors affecting drug delivery efficiency and therapeutic efficiency. Here, we demonstrated the effect of lipid concentration and mixing performance on the LNP size using microfluidic devices with the aim of understanding the LNP formation mechanism and controlling the LNP size precisely. We fabricated microfluidic devices with different depths, 11 μm and 31 μm, of their chaotic micromixer structures. According to the LNP formation behavior results, by using a low concentration of the lipid solution and the microfluidic device equipped with the 31 μm chaotic mixer structures, we were able to produce the smallest-sized LNPs yet with a narrow particle size distribution. We also evaluated the mixing rate of the microfluidic devices using a laser scanning confocal microscopy and we estimated the critical ethanol concentration for controlling the LNP size. The critical ethanol concentration range was estimated to be 60–80% ethanol. Ten nanometer-sized tuning of LNPs was achieved for the optimum residence time at the critical concentration using the microfluidic devices with chaotic mixer structures. The residence times at the critical concentration necessary to control the LNP size were 10, 15–25, and 50 ms time-scales for 30, 40, and 50 nm-sized LNPs, respectively. Finally, we proposed the LNP formation mechanism based on the determined LNP formation behavior and the critical ethanol concentration. The precise size-controlled LNPs produced by the microfluidic devices are expected to become carriers for next generation nanomedicines and they will lead to new and effective approaches for cancer treatment.

## Introduction

Lipid nanoparticles (LNPs) or liposomes are the most widely used drug carriers for nanomedicines [1–3]. LNPs allow two targeting modes: passive targeting by the enhancement permeability and retention (EPR) effect, and active targeting using surface modification with ligands. In addition, LNPs are able to encapsulate a variety of materials such as low molecular compounds [4, 5], gold nanoparticles [6], peptides [7], DNA [8, 9], and RNA [10, 11]. These features make it possible to achieve high flexibility in the design of LNP-based nanomedicines and LNPs have been reported to achieve good therapeutic effects [12–15]. The nanomedicine size is also considered to be a significant factor influencing the therapeutic effects, because many large-sized nanomedicines is trapped and filtered out by the mesh-like structures of the spleen. On the other hand, nanomedicines smaller than 10 nm are removed by the lymphatic system. Suitably sized nanomedicine can effectively accumulate in target organs and produce high therapeutic effects.

Recently, the size dependency of nanomedicines on the penetration efficiency in tumor tissues has attracted attention for the development of the next generation nanomedicines [16, 17]. In the case of micelle-based nanoparticles, 30 nm-sized micelles showed higher penetration efficiency than 70 nm-sized micelles did [16]. We also reported the effect of the LNP size on the penetration efficiency into tumor tissues in animal tests [17]. We intravenously administrated 40 and 70 nm-sized LNPs encapsulating siRNA to ICR mice, and then we evaluated the intrahepatic distribution of siRNA. Although both sizes of LNPs showed gene silencing activity, 40 nm-sized LNPs were able to deliver siRNA to the hepatocytes more effectively. Therefore, precise control of particle size is desired for the development of the next generation nanomedicines; such size-controlled nanomedicines can be expected to realize particle-size-dependent drug delivery systems. However, the precise size control of LNPs, for example, the LNP size tuning at 5–10 nm intervals is difficult by the conventional LNPs preparation method.

Microfluidic-based techniques are expected to be excellent methodologies for not only LNP synthesis but also extracellular vesicles separation including exosomes [18–28]. The LNPs are easily produced by injection of organic solutions containing lipids and aqueous solutions into a microfluidic device. Typically, the LNP size is controlled by the flow rate of the solutions and the flow rate ratio (FRR: the flow rate of the aqueous solution to the flow rate of the lipid solution), and rapid mixing is also a significant factor for controlling the LNP size and producing small-sized LNPs. To enhance the mixing efficiency, chaotic mixer structures have been employed in microfluidic devices [20, 23, 24], and 20 nm-sized LNPs were formed by applying an extremely high flow rate condition for the microfluidics. However, the mixing performance of the chaotic mixer under the high flow rate condition is decreased due to the fluid dynamics [29, 30]. We therefore previously investigated the effect of the chaotic mixer on the LNP size and their size distributions. We found that the complete mixing of solutions, which were saline and the lipid/ethanol solutions, was not necessary for controlling the LNP size and producing the small-sized LNPs [20]. In addition, we proposed a LNP formation mechanism in the microfluidic device and a concept of critical concentration to control the LNP size. In brief, the LNPs are formed by the following processes in the microfluidic device: aggregation of lipid molecules, formation of intermediate disk-like structures, fusion of these intermediate disk-like structures, and transformation of the intermediate disk-like structures to enclose LNPs. The intermediate disk-like structures could be formed and were stable at the critical concentration and the lifetime of the intermediate disk-like structures dominated the LNP size. In spite of the indispensability of the critical concentration for precise size tuning of LNPs, the critical concentration range is not well understood. Understanding the LNP formation mechanism and the critical concentration allows us to develop high performance LNPs production systems using microfluidics, leading to the next generation LNPs-based nanomedicines.

In this study, we investigated the effect of lipid concentration and the mixing performance of microfluidic devices on the LNP formation behavior. We fabricated three types of microfluidic devices: the microfluidic device with different depths, 11 μm or 31 μm, of their chaotic micromixer structures and the microfluidic device without micromixers. The LNP size and the mixing rate were measured using the microfluidic devices by changing the flow rate conditions. The LNP formation mechanism and the critical concentration were discussed using the relationship between the formed LNP size and the dilution rate of ethanol.

## Experimental

### Materials

1-palmitoyl-2-oleoyl-sn-glycero-3-phosphocholine (POPC) and 1, 2- dioleoyl-*sn*-glycero-3-phosphocholine (DOPC) were purchased from the NOF Corporation (Tokyo, Japan). 1,2-dioleoyl-*sn*-glycero-3-phsphoethanolamine-N-(lissamine rhodamine B sulfonyl) (Rho-PE) was purchased from Avanti Polar Lipids, Inc. (810150C, Alabaster, AL, USA). Ethanol, sodium chloride, and chloroform were purchased from Wako Pure Chemical Industries, Ltd. (Osaka, Japan).

### Fabrication of the microfluidic devices

The master molds of the microfluidic devices with chaotic micromixers were fabricated by two-step photolithography [27]. The master molds were made from SU-8 3010 and 3050 (Nippon Kayaku Co., Ltd., Tokyo, Japan). First, SU-8 3050 was poured onto 3-in silicon wafers (SUMCO Co., Tokyo, Japan) to make the first SU-8 layer. The silicon wafers were spin-coated using a spin coater (MS-A100, Mikasa Shoji, Co., Ltd., Tokyo, Japan) to a thickness of 79 μm and then the wafers were baked on a hot plate for 30 min to evaporate the solvent. These pre-baked silicon wafers were exposed to UV light with a mask aligner (M-1S, Mikasa Shoji) through photomasks (12700 dpi, Unno Giken Co., Ltd., Tokyo, Japan). Then, the silicon wafers were post-baked on the hot plate and SU-8 3010 or 3050 was poured again onto the wafers to make the second SU-8 layer. These silicon wafers were then spin-coated to a thickness of 11 or 31 μm and they underwent a second pre-baking. Photomasks for the second SU-8 layer (the chaotic micromixer part) were aligned and exposure to UV light was done. After the post-baking and developing processes, the SU-8 molds were treated with a vapor of trichloro (1*H*,1*H*,2*H*,2*H*-perfuluorooctyl) silane (Sigma-Aldrich, St. Louis, MO, USA). Polydimethylsiloxane (PDMS; SILPOT 184 W/C, Dow Corning Toray Co., Ltd., Tokyo, Japan) was cast onto the SU-8 mold and cured in an oven at 70°C for 1 h. The PDMS replica was cut out from the SU-8 mold and holes for inlets and the outlet were punched out. The PDMS replica was bonded to a glass substrate (S1111, Matsunami Glass Ind., Ltd., Osaka, Japan) using an oxygen plasma treatment apparatus (CUTE-1MP/R, Femto Science, Gwangju, Korea). Poly(etheretherketone) (PEEK) capillaries (inner diameter = 300 μm, outer diameter = 500 μm) were purchased from the Institute of Microchemical Technology Co., Ltd. (Kanagawa, Japan). PEEK capillaries were connected to the inlets and the outlet of the microfluidic device and cured with superglue. The master mold of the microfluidic device without the micromixers was fabricated by the standard photolithography method and the microfluidic device was made by the same replica molding procedure as described above.

### Synthesis of the LNPs

**Fig 1:**
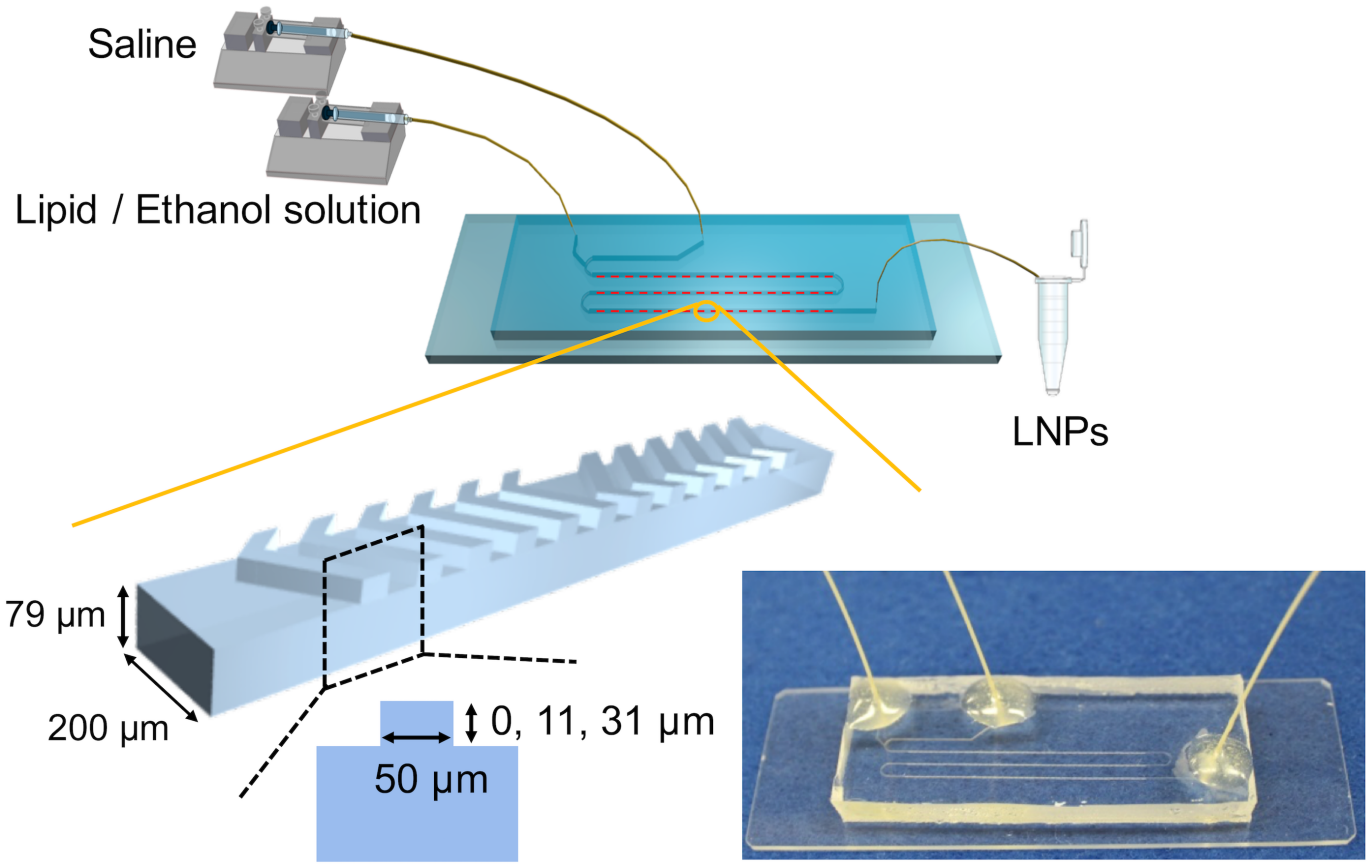
Schematic illustration and photograph of the experimental setup. The width and depth of the microchannel were 200 and 79 μm. The total length of microchannel was 110 mm. The heights of the chaotic mixer structures were 0, 11, and 31 μm, for the NC device (without micromixers), the CM_11 device, and the CM_31 device, respectively. An enlarged image shows one cycle of the chaotic mixer structures. The CM_11 and CM_31 devices were equipped with 69 cycles of chaotic mixer structures.

POPC was dissolved at 5, 10, or 20 mg/mL in ethanol. Saline was prepared by dissolving sodium chloride at 154 mM in ultrapure water (Direct-Q UV system, EMD Millipore Co., Billerica, MA). Fig 1 shows a schematic illustration of the experimental setup and the design of the microchannel structure. We used three types of microfluidic devices: the microfluidic device with different depths, 11 μm (the CM_11 device) or 31 μm (the CM_31 device), of their chaotic micromixer structures and the microfluidic device without micromixers (the NM device). The total length of microchannel was 110 mm and the CM_11 and CM_31 devices were equipped with 69 cycles of basic mixer structures. The width and space of the micromixers was 50 μm. Separate syringes (GASTIGHT 1002, Hamilton Inc., Reno, NV, USA) were filled with the lipid solution and saline and the syringes were connected to the microfluidic device. We used syringe pumps (Model 100, BAS Inc., Tokyo, Japan) to feed the solutions into the microfluidic device. LNPs were continuously formed by mixing the solutions in the microfluidic device. LNP solution was collected in a microtube at the outlet of the PEEK capillary. The collected LNP solution was stored in a refrigerator until particle size measurement. The size of the LNPs was measured by dynamic light scattering (DLS) using a Zetasizer Nano ZS ZEN3600 instrument (Malvern Instruments, Worcestershire, UK).

### Evaluation of the mixing performance

A mixture of DOPC and Rho-PE dissolved in ethanol was employed as a lipid solution for evaluating the mixing performance. The concentration of lipid solution was 10 mg/mL DOPC containing 0.1 mol% of Rho-PE. DOPC and Rho-PE were dissolved in chloroform in a centrifuge tube and dry nitrogen gas was blown through to evaporate chloroform. To remove chloroform completely, the centrifuge tube was set in a desiccator and kept under vacuum by a rotary pump for overnight. After the chloroform was removed, an appropriate volume of ethanol was injected into the centrifuge tube and the lipid solution was strongly shaken several times. We measured the fluid dynamics in the microfluidic devices using a laser scanning confocal microscope (A1R, Nikon, Tokyo, Japan). Scanning region of the x-y plane was set at 512 × 256 pixels. Fluorescence images at a cross section of the microchannel were obtained at 5 μm step intervals (z-axis: depth of the microchannel) and scanning speed was set at 2 *f*/s. We evaluated the mixing performance of the three microfluidic devices, two with 11 μm or 31 μm depths of chaotic micromixer structures (CM_11 and CM_31) and the microfluidic device without chaotic micromixers (NM). The scanning region of the z-axis was set at 100, 120, and 90 μm, for the CM_11, CM_31, and NM devices, respectively. The fluorescence images were analyzed using Image J (NIH) to obtain the gray scale color intensity. The mixing rate was calculated from the following equation [31]:

$$\text{Mixing}\;\text{rate}\;\left[\%\right]=\left(1-\frac{\sqrt{\frac{1}{N}\sum_{i=1}^{i=N}{\left(I_{i}-I_{i}^{Perf.\;mix}\right)}^{2}}}{\sqrt{\frac{1}{N}\sum_{i=1}^{i=N}{\left(I_{i}^{0}-I_{i}^{Perf.\;mix}\right)}^{2}}}\right)\;\times \;100$$ (1)

where, *N*, *I*_*i*_, *I*_*i*_^*0*^, and *I*_*i*_^*Perf*. *Mix*^ are the total number of pixels, the gray scale intensity at pixel *i*, the gray scale intensity at pixel *i* without mixing or diffusion, and the gray intensity of the completely mixed solution at pixel *i*, respectively. The 80% mixing rate was assumed as complete mixing.

## Results and discussion

### Effect of lipid concentration on LNP formation

**Fig 2:**
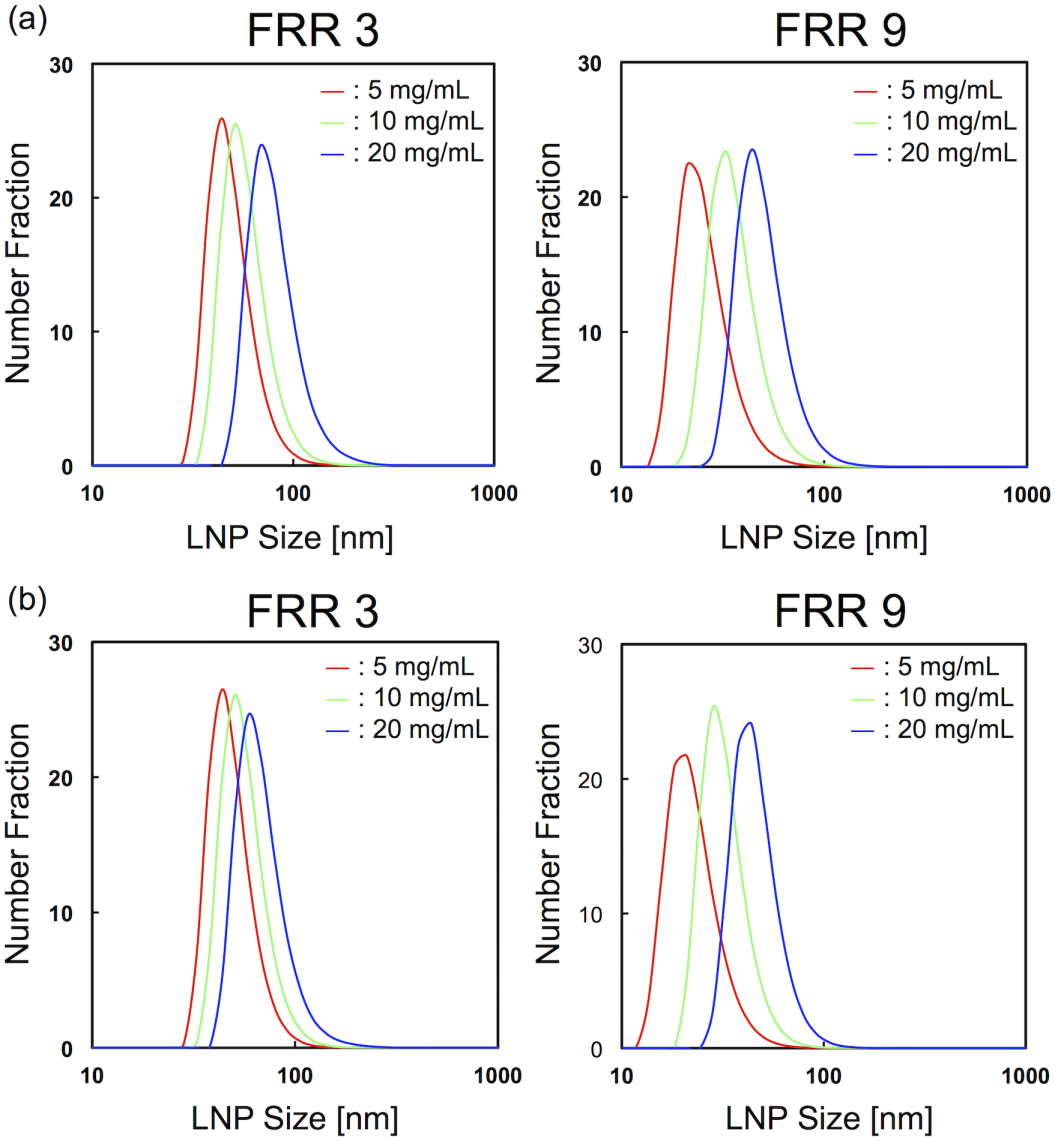
Size distributions of LNPs at FRR of 3 and 9 at different lipid concentration conditions. The flow rates were (a) 100 μL/min and (b) 500 μL/min.

**Fig 3:**
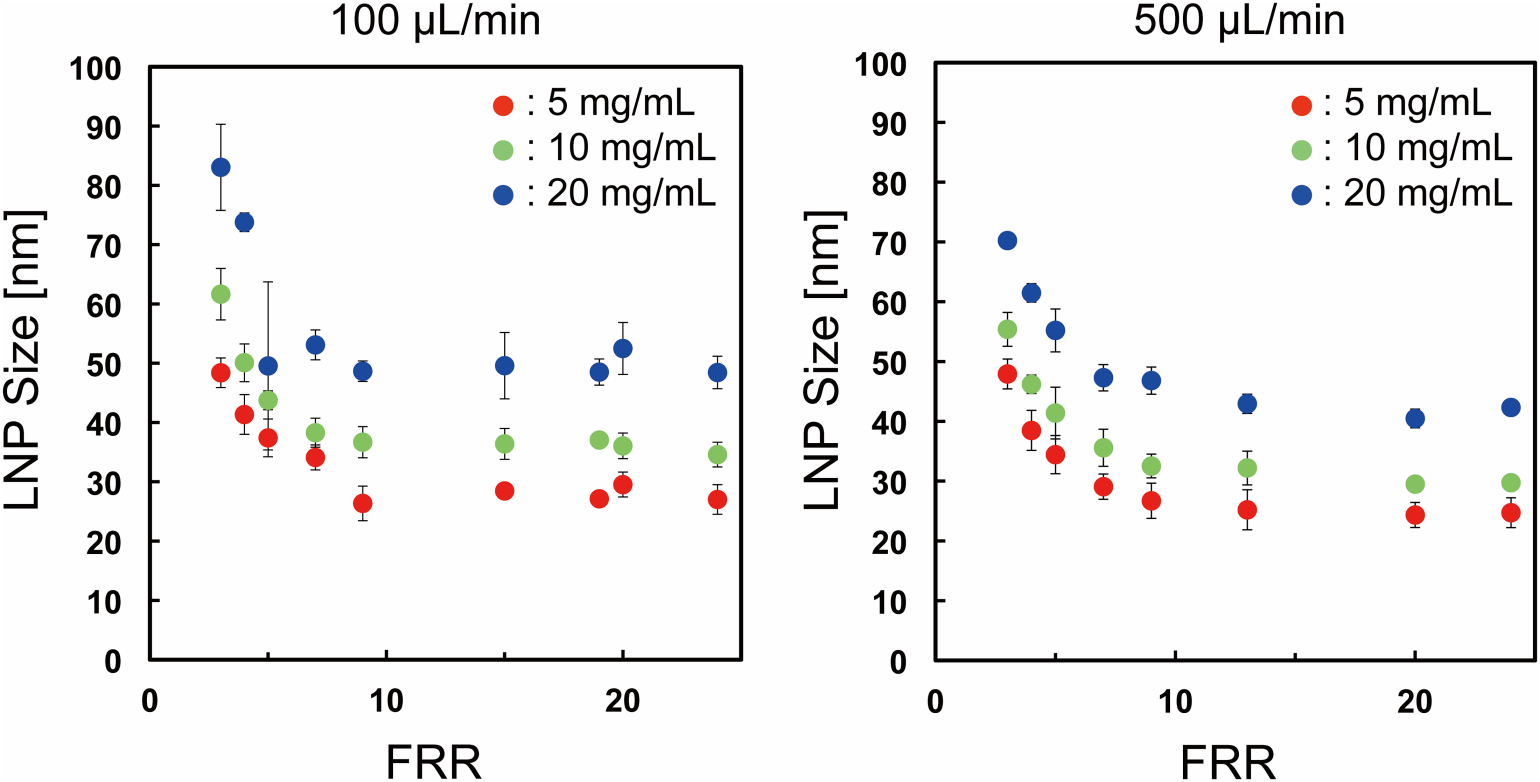
The effect of lipid concentration on LNP size under different flow conditions. The error bars represent the standard deviation calculated from repeating each LNP formation experiment at least three times.

First, we focused on the effect of lipid concentration on the LNP size. The CM_31 device was used for the experiment. Fig 2(a) shows the LNP size distributions at the flow rate of 100 μL/min and the flow rate ratio (FRR: the flow rate of the aqueous solution to the flow rate of the lipid solution) of 3 and 9. LNP sizes were increased with increasing lipid concentration. The smallest size of LNPs was produced by the 5 mg/mL POPC/ethanol solution, regardless of the FRR. Then, we changed the flow rate to 500 μL/min, because the flow rate condition affected the LNP size. Fig 2(b) shows the LNP size distributions at the flow rate of 500 μL/min and the FRR of 3 and 9. We observed similar LNP formation behavior between 100 μL/min and 500 μL/min. Low lipid concentration or high FRR condition was able to form small-sized LNPs. We summarize the effect of lipid concentration on LNP size under the different FRRs in Fig 3. When we used 5 mg/mL POPC/ethanol as a lipid solution, the LNPs formed at 100 μL/min were almost the same size as those of the LNPs formed at 500 μL/min. On the other hand, 10 and 20 mg/mL lipid solutions produced slightly larger size LNPs at 100 μL/min than those formed at the flow rate of 500 μL/min. The LNP size differences by changing the flow rate were approximately 5 nm and 10 nm, for 100 μL/min and 500 μL/min, respectively. We also calculated the particle size polydispersity index (PDI) to evaluate the uniformity from the particle size distribution. The PDI values were mainly smaller than 0.1 as shown in S1 Fig. These results suggest that the lipid concentration is one of the essential factors for controlling the LNPs size. We were able to produce 30 nm-sized LNPs at high FRR and flow rate conditions, which could penetrate in tumor tissues effectively, even though the silencing efficiency was slightly reduced compared with 70 nm LNPs [13]. In this study, the size of the LNPs was measured by DLS, because DLS is the most widely used LNP size evaluation method. To observe the actual LNP size, we also tried to measure the negatively stained LNPs by TEM. 20–30 nm LNPs were mostly observed by TEM analysis, although the some LNP shapes were deformed due to the dry out under the vacuum condition (data not shown). Consequently, LNP size tuning was precisely achieved by controlling the flow condition and the lipid concentration from 25 nm to 80 nm.

Previously, we proposed the LNP formation mechanism in the microchannel [20] that was based on reported [32–34] molecular dynamics (MD) simulations. Lipid molecules are gradually self-assembled by mixing aqueous and lipid solutions, because lipids are amphiphilic molecules and cannot be dissolved in aqueous solutions. The self-assembled intermediate disk-like structures are called bilayered phospholipid fragments (BPFs) and they are thermodynamically semi-stable [35, 36]. The BPFs grow and finally transform to enclose the LNPs. We found that rapid dilution by aqueous solution enables the formation of small-sized LNPs. Here, POPC molecules can dissolve due to the presence of ethanol. However, the mixing of lipid and aqueous solutions induces the hydration of ethanol and increases the solution polarity. When we used a high concentration of lipid solution, the BPFs frequently formed at the saline-ethanol interface. The BPFs fuse and grow to a limiting size, which is assumed to be semi-stable under the described conditions. A large amount of BPFs makes it possible to grow larger-sized LNPs due to the fusion of the BPFs. For this reason, the LNP size produced at the high lipid solution concentration is larger compared with the size produced at the low lipid solution concentration. We therefore assume that the dilution rate of ethanol affects the LNP size.

### Effect of mixing performance on the LNP formation

**Fig 4:**
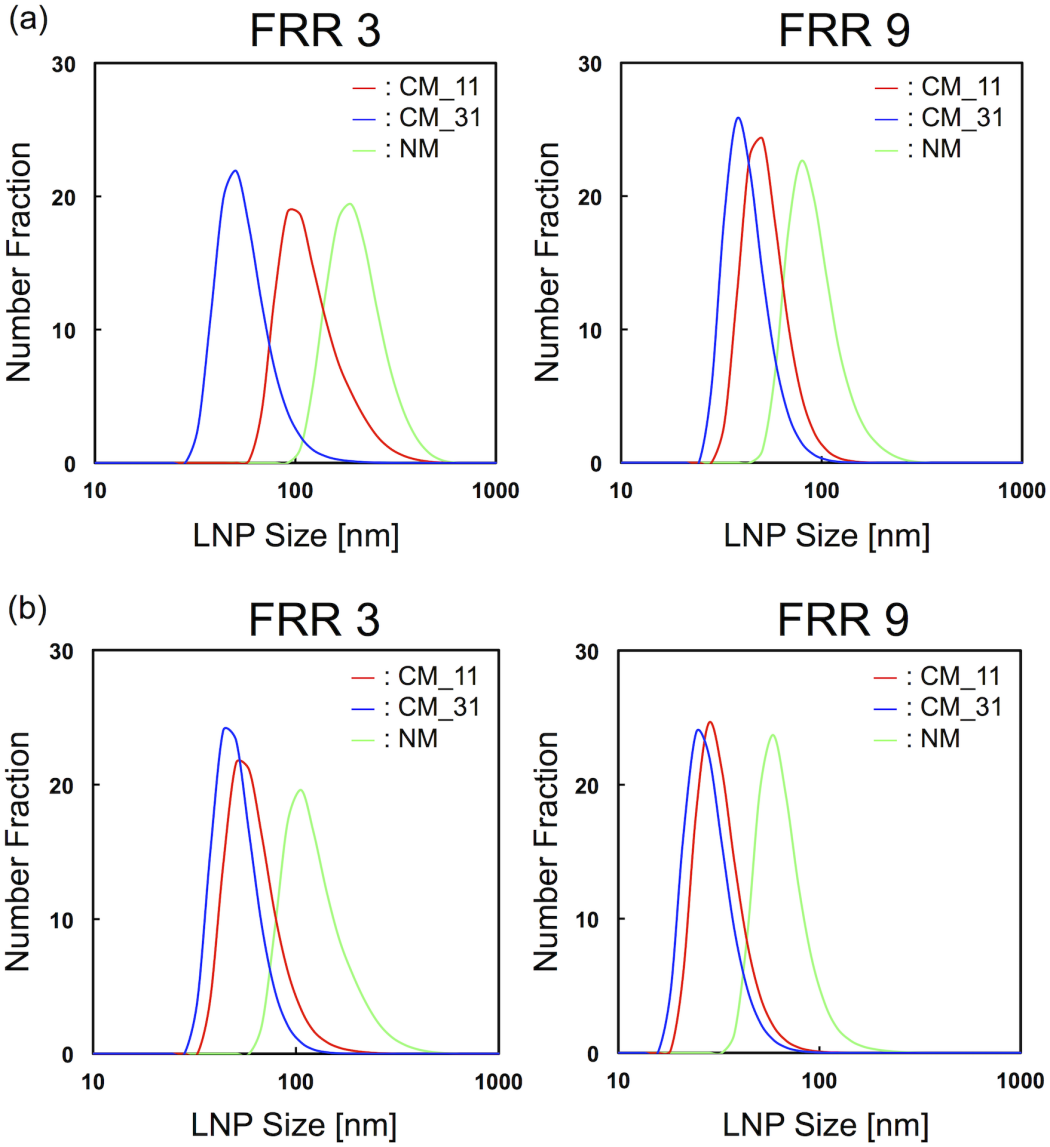
Size distributions of LNPs formed in the microfluidic devices at FRR of 3 and 9. The flow rates were (a) 50 μL/min and (b) 500 μL/min.

We fabricated three types of microfluidic devices, the CM 11, CM 31 and NM devices, and used them with the aim of elucidating the effect of mixing or the diluting rate of ethanol on the LNP size. The flow rate and the FRR were also changed to confirm the effect of dilution rate on LNP size. Fig 4(a) shows the LNP size distributions at the flow rate of 50 μL/min and the FRR of 3 and 9. The LNP size was dramatically changed by the micromixer structures. The CM_31 device was able to produce suitably sized LNPs with a diameter smaller than 100 nm, which is effective for drug delivery, regardless of the FRR condition. Conversely, the CM_11 device produced slightly larger LNPs with a diameter of 100 nm at FRR of 3. However, when we employed the FRR of 9 for LNP synthesis, the LNP size was almost the same size as that obtained with the CM_31 device. In addition, using the NM device, we obtained the larger LNP size and wider size distribution compared with the other devices. These results indicate that the rapid dilution of ethanol is essential for producing the small-sized LNPs. Fig 4(b) represents the LNP size distributions at the flow rate of 500 μL/min and the FRR of 3 and 9. For FRR of 3, the LNP size diameter difference obtained between the CM_11 and the CM_ 31 devices was from 5 to 10 nm and they could form small-sized LNPs, whereas the NM device could not form small-sized LNPs with a diameter less than 50 nm.

**Fig 5:**
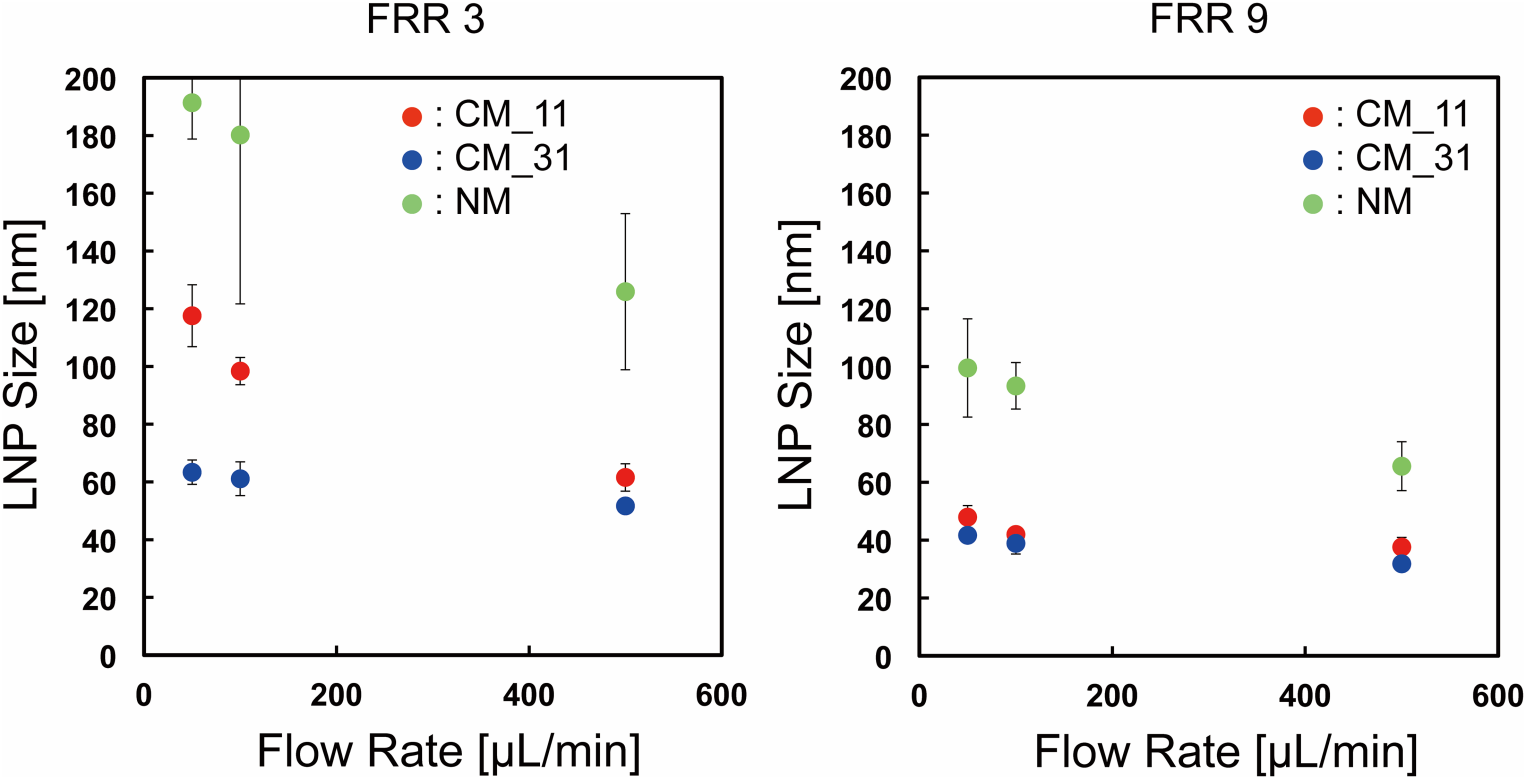
The effect of mixer structures on the LNP size at different flow conditions. The error bars represent the standard deviation calculated from repeating each LNP formation experiment at least three times.

Fig 5 summarizes our findings on the effect of the chaotic mixer structures on the LNP size at different flow conditions. The LNP size formed for FRR of 7 is shown in S2 Fig. These results also suggest that the chaotic mixer structures enhance the controllability of LNP size. The CM_31 device had the best LNP size controllability among the three microfluidic devices and the size of the LNPs obtained by the CM_31 device ranged from 30 to 60 nm. On the other hand, the CM_11 device enabled the production of a wide range of LNPs sized from 35 to 120 nm, although the LNP size distribution obtained was only slightly larger than that of the CM_31 device. At FRRs of 7 and 9, the CM_11 and CM_31 devices showed similar LNP formation behavior. The high FRRs of 7 and 9 make the rapid dilution of ethanol possible, because of the high ratio of aqueous phase. In addition, the amount of lipid molecules per unit volume was also smaller than that for the FRR of 3. Therefore, this result also indicates that the dilution rate plays an important role in formation of LNPs.

### Evaluation of mixing performance of the microfluidic devices

**Fig 6:**
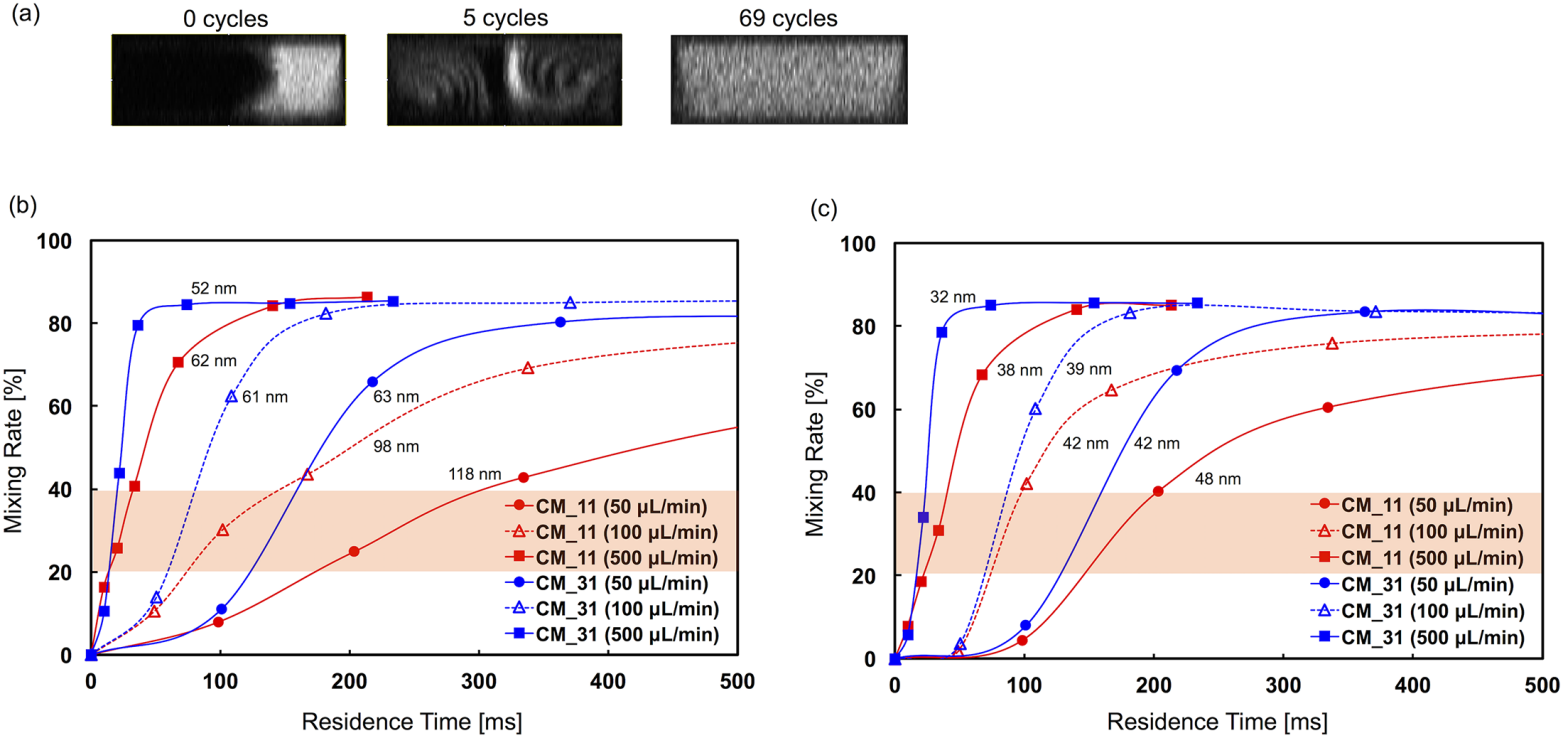
Evaluation of mixing performance of the microfluidic devices. (a) The confocal images of a cross section of the CM_31 device at different positions. The measurement positions of the images were before passing through the mixer structures (0 cycles), and after passing through the 5th and the 69th mixer structures. The flow conditions were 100 μL/min and FRR of 3. (b-c) Comparison of the mixing rate for 500 ms between the CM_11 and CM_31 devices at (b) FRR of 3 and (c) FRR of 9. The plots represent the mixing rate after passing through the 1st, 5th, 10th, 23th, 46th, and 69th chaotic mixer structures. The mixing rate in the entire microchannel is shown in S3 and S4 Figs.

We carried out a visualization experiment to evaluate the mixing performance of each microfluidic device. We measured fluorescence images at the merging point of the lipid solution and the saline, after passing through the 1st, 5th, 10th, 23th, 46th, and 69th chaotic mixer structures. The mixture of the headgroup labeled fluorescent lipid (Rho-PE) and POPC was used as the lipid solution. The fluorescence images were analyzed by image J and the mixing rate was calculated according to Eq (1). Fig 6(a) shows confocal images of a cross section of the CM_31 device at different positions. The flow conditions were 100 μL/min and FRR of 3. We confirmed that the solutions were mixed completely after passing through the 69th chaotic mixer structures. Fig 6(b) compares the mixing rate for 500 ms between the CM_11 and CM_31 devices at FRR of 3. The mixing rate in the entire microchannel is shown in S3 and S4 Figs. The x-axis represents the residence time from the merging of the lipid and the saline at the inlet of the microchannel. The mixing performance of the CM_31 device was higher than that of the CM_11 device. Notably, for the flow rates of 50 and 100 μL/min in the CM_11 device, the mixing performance dramatically declined compared with the CM_31 device and large-sized LNPs were formed due to the slow dilution rate. As demonstrated in Fig 5, for the CM_11 device the LNP size ranged from 60 to 120 nm, indicating dependency on the flow rate at FRR of 3, whereas for the CM_31 device there was no dependency (size: 50–60 nm). Large-sized LNPs formed under the slow dilution rate condition. Moreover, for the CM_31 device, 60 nm-sized LNPs formed at 50 and 100 μL/min, although the times necessary to reach the 20% mixing rate were different. These results suggest that the rapid mixing at a mixing rate exceeding 20% is critical for producing the small-sized LNPs.

Complete mixing is not required for LNP synthesis using the microfluidic device, because 60 nm sized LNPs formed for the 50 μL/min flow rate even though a long mixing time (> 400 ms) was needed for complete mixing (S3 and S4 Figs). For the optimal flow condition of the chaotic mixer, the Reynolds number should be from 1 to 100 from the viewpoint of fluid dynamics [29, 30]. Therefore the mixing performance of the 500 μL/min condition (40% mixing at the 5th chaotic mixer structures) slightly declined compared to that of the 50 and 100 μL/min conditions (60–70% at the 5th chaotic mixer structures). However, the high flow rate condition enables rapid passing through many chaotic mixer structures at unit time. For this reason, the high flow rate condition was able to produce the smallest LNPs among all flow conditions.

Fig 6(c) compares the mixing rate for 500 ms between the CM_11 and CM_31 devices at FRR of 9. The mixing performance of the CM_11 device was improved compared to its performance at FRR 3. The LNP size diameter difference obtained between the CM_11 and CM_31 devices at the same flow rate condition was almost 5 nm (Fig 5). Here, we focused on the 50 and 100 μL/min conditions to elucidate the critical mixing rate or the critical ethanol concentration for producing small-sized LNPs. At both flow rates, the mixing rate increased after passing through the 1st chaotic mixer. However, the mixing rate after passing through the 5th chaotic mixer was calculated to be higher than 40% for the CM_11 device, regardless of the flow rate. After passing through the 5th chaotic mixer, the mixing performance declined and then the mixing rate increased to 60% at the 10th chaotic mixer. On the other hand, the mixing rates of the CM_31 device at the 5th chaotic mixer were calculated to be 70% and 60% for the flow rates of 50 and 100 μL/min, respectively. However, 48 nm-sized LNPs were formed at 50 μL/min in the CM_11 device, and 40 nm-sized LNPs were formed at the other conditions. In other words, the LNP size was almost the same at these conditions. From these results, we assume that rapid mixing from 20% to 40% is the critical factor for producing the small-sized LNPs and controlling the LNP size. In the case of FRR of 9, the slope of the mixing rates between 20% to 40% for each flow rate conditions were roughly calculated to be 0.4–2% /ms from the Fig 6(c). This suggests that the formation of LNPs was achieved by mixing on the 10, 15–25, and 50 ms time-scales for 30, 40, and 50 nm-sized LNPs at the FRR of 9, respectively.

### LNP formation process

**Fig 7:**
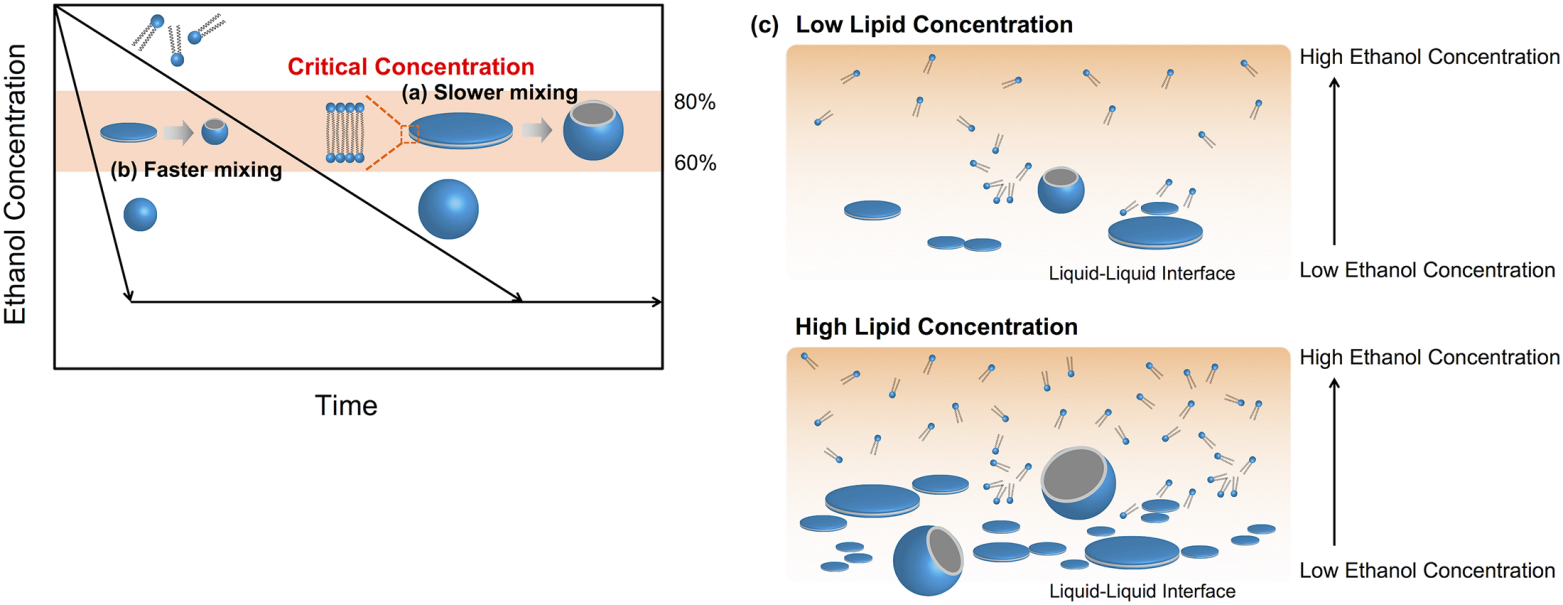
Summary of the LNP formation process in the microfluidic device. LNP formation process in a microfluidic device at (a) slower mixing and (b) faster mixing. (c) Schematic illustration of the LNP formation at the saline-ethanol interface.

A summary of the LNP formation process in the microfluidic device is shown in Fig 7. We focused on the dilution rate of ethanol and the concentration of lipid based on the BPF formation process. A hydrophobic chain of lipids is self-assembled due to the increasing solution polarity. The BPFs, which are semi-stable, grow until they are transformed into stable closure vesicles (that is, the LNPs) as shown in Fig 7(a). The growth of BPFs induces the increase of surface energy in the reaction system. When the ethanol concentration in the neighborhood of the BPFs is moderate, the BPFs are able to grow until they reach their thermodynamically stable size. Then, the grown BPFs are transformed to LNPs to decrease the surface energy in the reaction system. When ethanol around the BPFs is diluted rapidly, the BPFs cannot grow enough to form large-sized LNPs (Fig 7(b)).

First, we estimated the apparent ethanol concentration from the following equation.

$$\text{Apparent}\;\text{critical}\;\text{ethanol}\;\text{concentration}\;\left[\%\right]=100-\;\frac{\left(1-\frac{1}{\text{FRR}\;+\;1}\right)\;\times \;100}{80}\;\times \;\text{mixing}\;\text{rate}$$ (2)

Briefly, the ethanol concentrations at the complete mixing condition were calculated to be 25% and 10% ethanol for the FRR of 3 and 9, respectively. From the experimental results and the hypothesis of the LNP formation process, we assume that the BPFs begin to form at the 80% ethanol condition (mixing rate: 20%) and to transform to LNPs at the 60% ethanol condition (mixing rate: 40%). We therefore consider that the ethanol concentration of 60 to 80% is critical for producing the small-sized LNPs and controlling the LNP size in this experimental system. The critical concentration could change according to the experimental conditions, such as the concentrations and types of lipids, solvents, and additives. Although the mixing rate or apparent ethanol concentration is considered as an index value of uniformity of the solution, the LNP size is affected by not only the mixing performance of the device but also the lipid concentration and FRR. The BPFs and the LNPs are formed at the saline-ethanol interface where the hydration of ethanol molecules and the aggregation of lipid molecules trigger the LNP formation. Therefore, we consider that complete mixing is not necessary for controlling LNP size. For example, 70 nm-sized LNPs, with only a small size deviation, could be produced for the flow rate of 500 μL/min and FRR of 9 using the NM device. In the microchannel, the concentration gradient of ethanol was formed at the saline-ethanol interface. From the viewpoint of fluid dynamics, the distance necessary to mix the solutions at the high flow rate is longer than that of the low flow rate. In other words, a narrow concentration gradient forms in the microchannel for the high flow rate even though FRR may be the same. In fact, we confirmed that the mixing rate of the high flow rate at the outlet of the microfluidic device was smaller than that of the low flow rate (data not shown). In this case, the hydration of ethanol is dominated by molecular diffusion at the saline-ethanol interface. The narrow concentration gradient makes it possible to form small-sized LNPs with the low LNP size distribution, because the BPFs only grow at the critical ethanol concentration. The high FRR condition also forms the narrow concentration gradient at the liquid-liquid interface. Moreover, the micromixer structures of the microfluidic device are able to accelerate attaining homogeneity of the solutions by increasing the liquid-liquid interface area. Increasing the liquid-liquid interface area and having a shorter diffusion distance compared to the NM device allowed the rapid dilution of ethanol. For these reasons, we consider that the rapid dilution of ethanol at the liquid-liquid interface is essential, and not complete mixing.

The high FRR condition or the concentration of lipids also affected the LNP size. In the case of the high FRR, the amount of lipid molecules at the saline-ethanol interface was lower than that of the low FRR. Therefore, the high FRR is considered to have the same effect as the low lipid concentration condition and the production of 30–40 nm-sized LNPs is enabled using the CM_31 device. On the other hand, the high concentration of lipids produced large-sized LNPs, regardless of the flow condition, as shown in Fig 3. The lipid concentration does not affect the mixing rate or the dilution rate of ethanol. Thus, the BPFs frequently form at the liquid-liquid interface under the high lipid concentration condition (Fig 7(c)). This BPF formation behavior is similar to that in the crystal nucleation process. The BPFs grow by fusion with individual small-sized BPFs followed by bending of the BPFs to form the enclosed LNPs [34]. The growth rate of BPFs depends on the concentration of BPFs and the dilution rate of ethanol. At the high concentration of BPFs, it is easy to fuse each BPF and then large-sized LNPs are formed. The transformation rate from the BPFs to the closed form was estimated to take 100–200 ns by MD simulations [32–34]. However, the BPFs growth process takes a longer time compared with the transformation process and includes the following processes: aggregation of lipid molecules, formation of BPFs, diffusion of BPFs, and fusion of BPFs. We therefore assume that the LNP size is dominated by the BPF growth process and the rapid dilution that is possible by using the microfluidic device offers a promising approach for precise control of LNP size.

## Conclusion

To summarize, we demonstrated the effect of lipid concentration and dilution rate on the LNP size using microfluidic devices. Having a low concentration of lipid solution and using the microfluidic device equipped with chaotic micromixer structures made it possible to produce small-sized LNPs with a narrow particle size distribution. We found that mixing performance of the microfluidic devices was the essential factor for producing the small-sized LNPs at the low flow rate or the low FRR. In addition, we proposed the LNP formation mechanism based on the fluid dynamics and assumed that the critical concentration of ethanol was 60–80% for controlling the LNP size. Ten nanometer-sized tuning of LNPs was achieved by the optimum residence time at the critical concentration and rapid dilution using the microfluidic devices with chaotic micromixer structures. The residence times at the critical concentration necessary to control the LNP size were 10, 15–25, and 50 ms time-scales for 30, 40, and 50 nm-sized LNPs, respectively. The critical concentration might be changed by experimental conditions. The properties of lipids such as their charge, and the size of hydrophobic or hydrophilic groups are also considered as important factors influencing LNP formation. However, we can estimate the critical concentration via the dilution rate, the lipid concentration and the properties of lipids, and easily adjust the appropriate synthesis condition to control LNP size precisely. We believe that the proposed LNP formation mechanism offers significant information for the development of novel microfluidic devices with good potential the practical applications. The precise size-controlled LNPs produced by the microfluidic devices are expected to be carriers for next generation nanomedicines and they will lead to new effective approaches for cancer treatment.

## Supporting information

S1 Fig Relationship between the PDI values and the lipid concentrations. **T**he particle size polydispersity index (PDI) was calculated by the measurement of dynamic light scattering. The flow rates were 100 and 500 μL/min and the FRRs were set at 3 to 49. The error bars represent the standard deviation calculated from repeating each LNP formation experiment at least three times.
(TIFF)

S2 Fig The LNP size formed in the three types of microfluidic devices at FRR of 7. The error bars represent the standard deviation calculated form repeating each LNP formation experiment at least three times.
(TIFF)

S3 Fig The mixing rate in the microfluidic devices at FRR of 3. (TIFF)

S4 Fig The mixing rate in the microfluidic devices at FRR of 9. (TIFF)
