# Supplementary figures and images for: Understanding the formation mechanism of lipid nanoparticles in microfluidic devices with chaotic micromixers
Source: PLoS One. 2017 Nov 28;12(11):e0187962. doi: 10.1371/journal.pone.0187962 (PMC5705116; doi:10.1371/journal.pone.0187962)

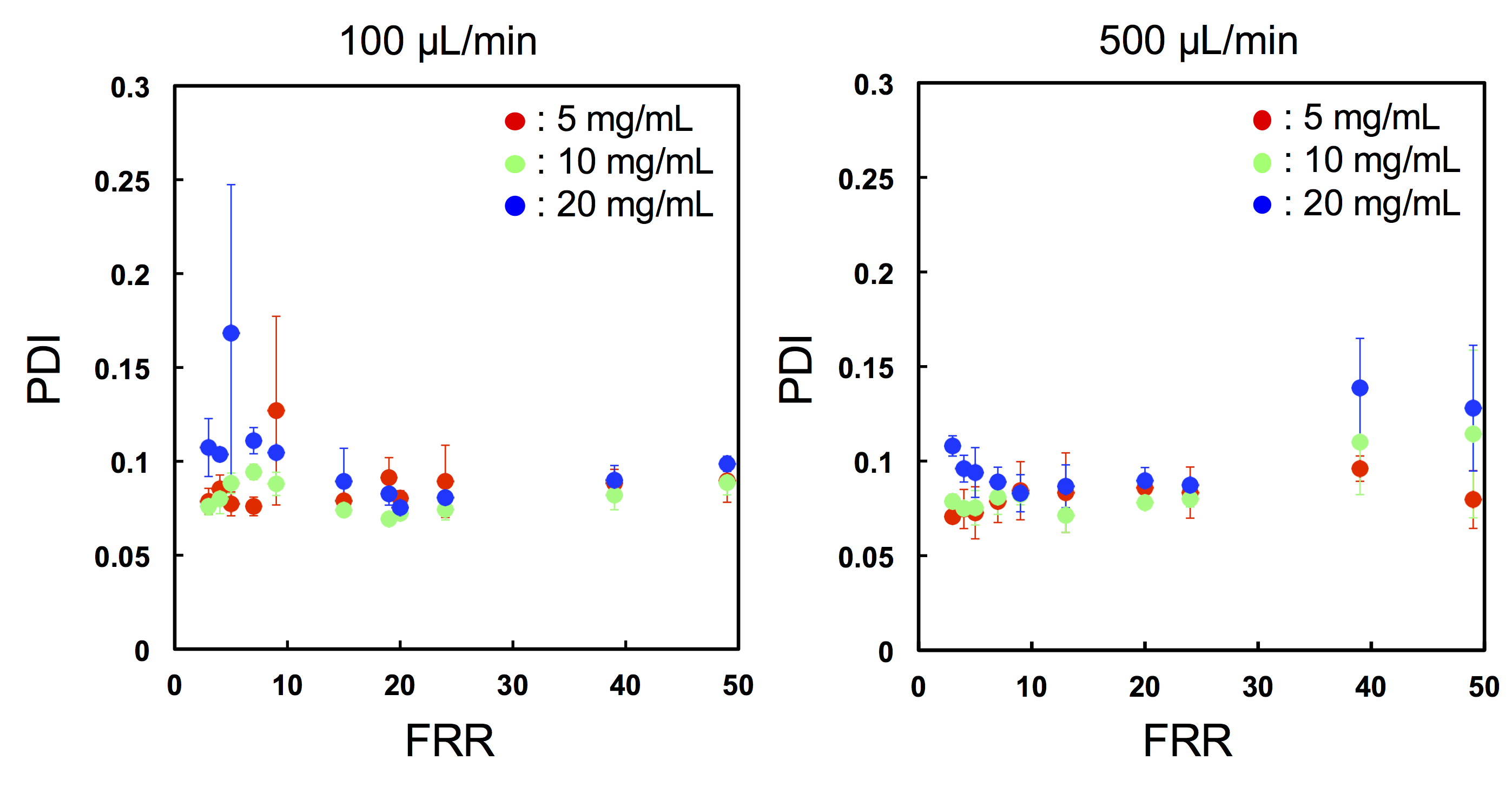

Supplement: S1 Fig — The particle size polydispersity index (PDI) was calculated by the measurement of dynamic light scattering. The flow rates were 100 and 500 μL/min and the FRRs were set at 3 to 49. The error bars represent the standard deviation calculated from repeating each LNP formation experiment at least three times. (TIFF) [file pone.0187962.s001.tiff]

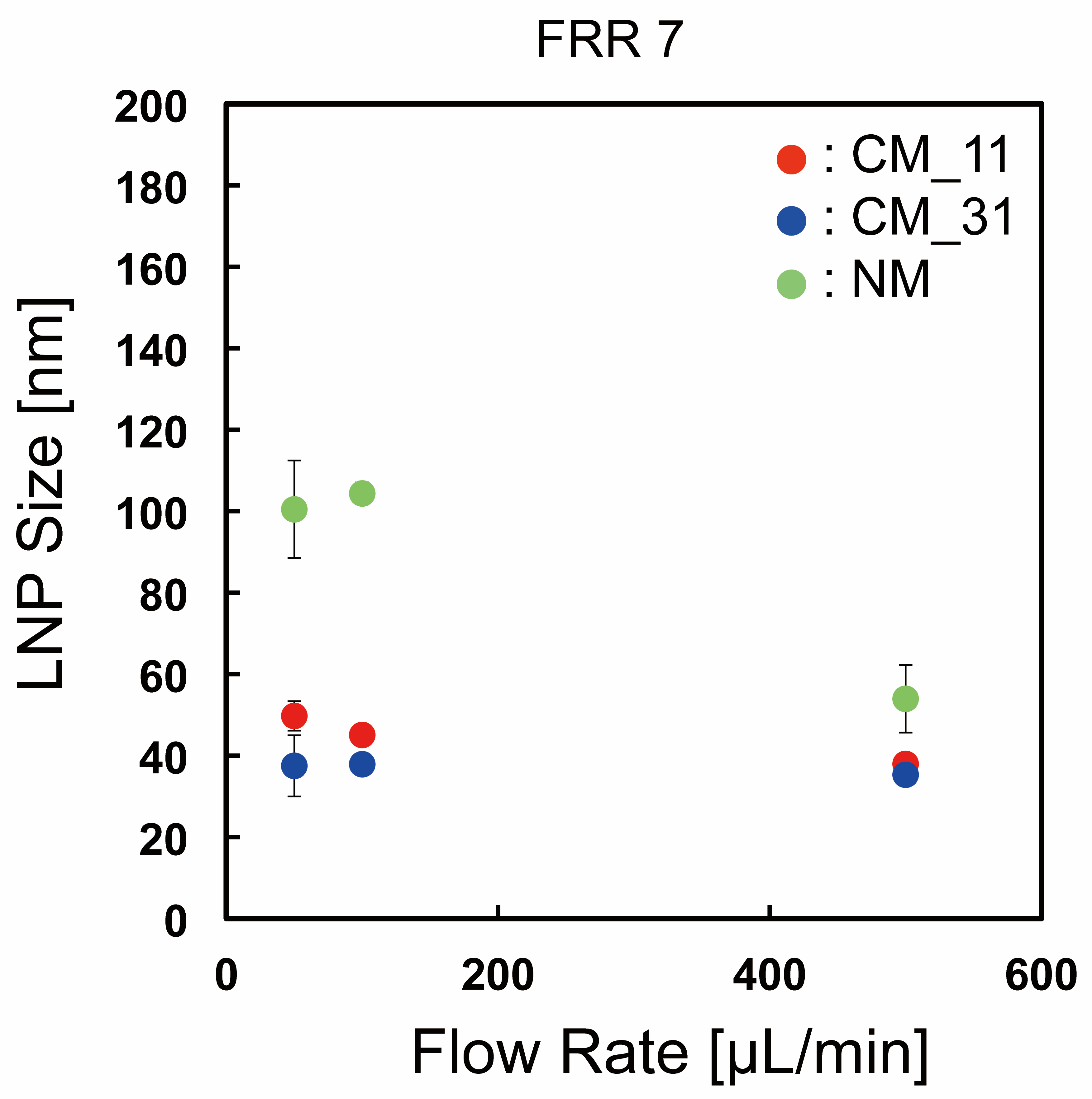

Supplement: S2 Fig — The error bars represent the standard deviation calculated form repeating each LNP formation experiment at least three times. (TIFF) [file pone.0187962.s002.tiff]

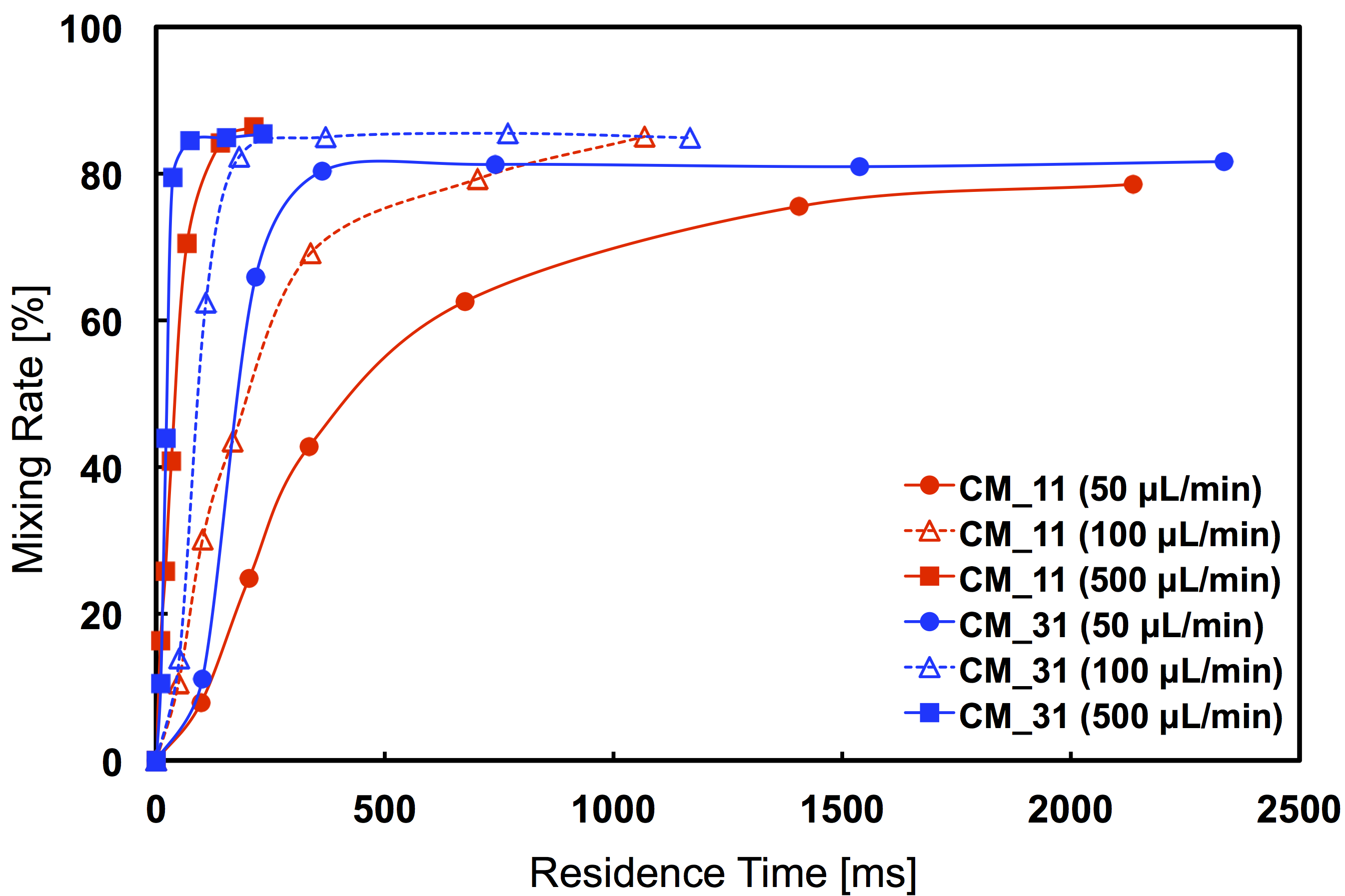

Supplement: S3 Fig — (TIFF) [file pone.0187962.s003.tiff]

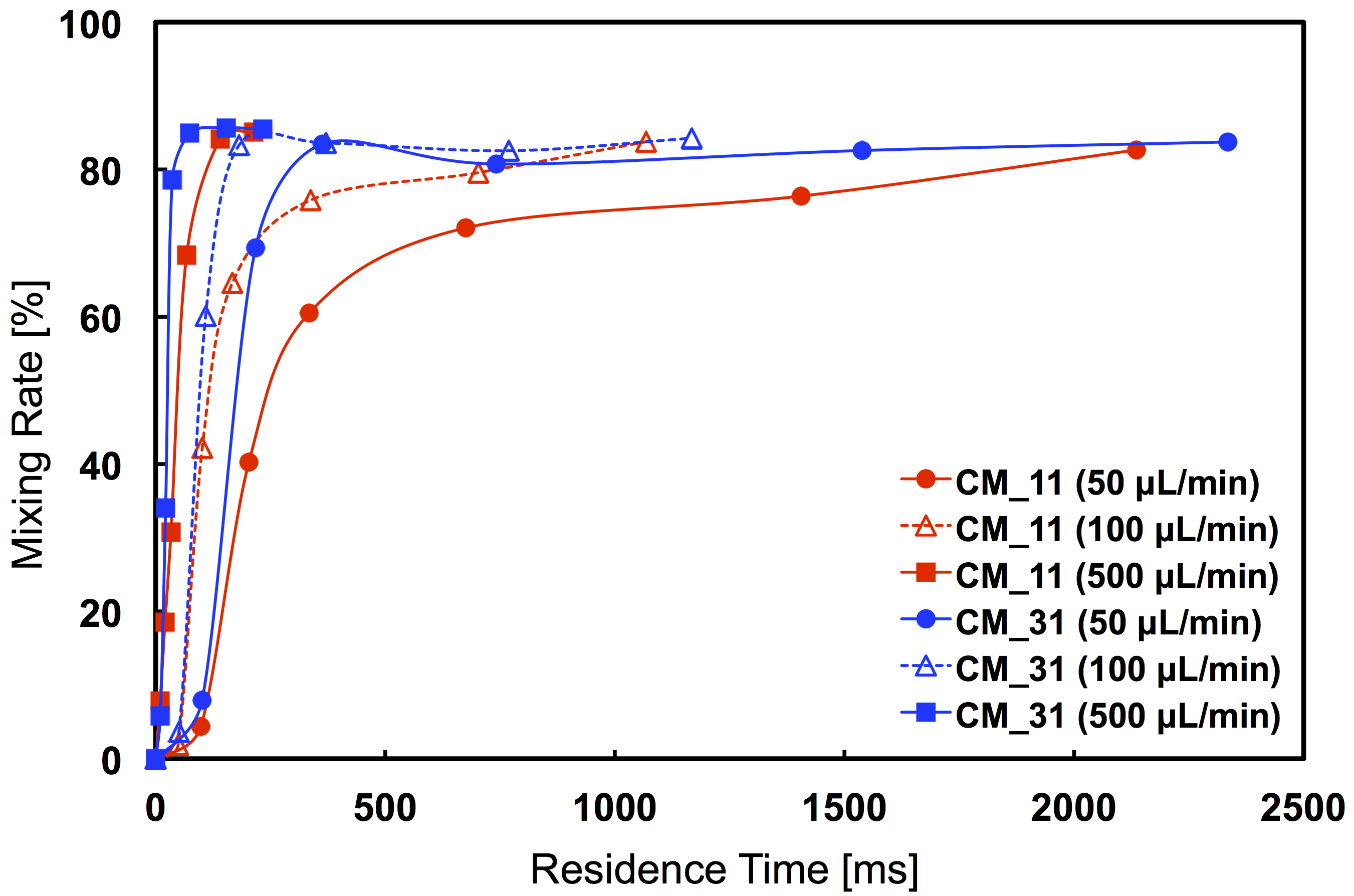

Supplement: S4 Fig — (TIFF) [file pone.0187962.s004.tiff]
